# Trained immunity in acute and chronic neurological diseases

**DOI:** 10.7554/eLife.106037

**Published:** 2026-01-15

**Authors:** Sijia Zhang, Arthur Liesz

**Affiliations:** 1 Institute for Stroke and Dementia Research (ISD), University Hospital, LMU Munich Munich Germany; 2 Munich Cluster for Systems Neurology (SyNergy) Munich Germany; Queen's University Canada; The University of Tokyo Japan

**Keywords:** trained immunity, brain disorders, innate immunity, stroke, alzheimer's disease, parkinson's disease

## Abstract

Trained immunity, the long-term reprogramming of innate immune cells to elicit an enhanced response upon subsequent challenges, has become a key concept in understanding a wide range of pathologies, including both acute and chronic inflammatory disorders. Recent evidence suggests that trained immunity also plays a significant role in the development and progression of various neurological disorders and related comorbidities, in which brain pathology can lead to trained immunity. This review summarizes the current understanding of trained immunity within both brain-resident immune cells and myeloid-derived innate immune cells, focusing on their roles in neurological disorders, such as ischemic brain injury, Parkinson’s disease, and Alzheimer’s disease. Additionally, we explore the heterogeneity of trained immunity across different conditions and its potential applications in clinical neurology.

## Introduction - basic concepts of trained immunity

Recent research has demonstrated that immune memory is not exclusive to adaptive immune system. Innate immune cells, including monocytes, macrophages, and dendritic cells, as well as hematopoietic stem and progenitor cells (HSPCs) and innate-like lymphocytes, such as natural killer (NK) cells, also exhibit memory-like behavior, known as trained immunity (TRIM) (Kaufmann et al., 2018; Netea et al., 2011). This phenomenon refers to that upon exposure to specific stimuli, innate immune cells undergo functional reprogramming, thus enabling an enhanced response upon secondary challenges (Netea et al., 2020). Compared to the typical antigen-specific immunological memory of the adaptive immune system, trained innate immunity shows less specificity and allows to respond to a broader range of pathogens (Kleinnijenhuis et al., 2012; Sun et al., 2009). Conversely, innate immune cells can also acquire a state of immune tolerance, in which prior stimulation—such as endotoxin exposure—dampens their responsiveness to subsequent challenges, representing an opposing form of innate immune memory (Biswas and Lopez-Collazo, 2009; Divangahi et al., 2021).

Recently, TRIM of the innate immune cells have been observed across various models, including external stimuli, such as microbial infections and vaccine administration (Schrum et al., 2018; Walk et al., 2019), and acute or chronic sterile inflammation—such as organ transplantation, brain ischemia, and Western diet (Braza et al., 2018; Christ et al., 2018; Simats et al., 2024). Notably, certain substances have been reported that can induce long-term reprogramming of the innate immune cells following transient stimulation. Pathogen-associated molecular patterns (PAMP) were first demonstrated to be the inducers of TRIM, typically released during microbial infection, such as β-glucans, a fungus-derived dectin-1 ligand (Netea et al., 2011). Recently, damage-associated molecular patterns (DAMPs) were also found to induce TRIM. Molecules, such as extracellular heme, vimentin, oxidized low-density lipoprotein (oxLDL), and high-mobility group box 1 (HMGB1), which are released during tissue damage, have been reported to reprogram HSPCs or mature myeloid cells (Bekkering et al., 2014; Braza et al., 2018; Jentho et al., 2021).

Growing evidence highlights the essential role of the interleukin (IL) family of cytokines, produced by immune cells in response to both PAMPs and DAMPs (Kaneko et al., 2019), in enabling innate immune cells to acquire training features upon stimulation. For instance, pre-activation with IL-12, IL-15, and IL-18 could generate a memory-like phenotype in isolated human NK cells (Leong et al., 2014). Additionally, IL-1 signaling has been shown to be crucial for the β-glucan-induced training of bone marrow HSPCs, providing protection against subsequent infections (Ciarlo et al., 2020; Mitroulis et al., 2018; Moorlag et al., 2020a; Teufel et al., 2022). Our research has further demonstrated the critical function of IL-1β in mediating TRIM in HSPCs following sterile systemic inflammation, such as ischemic stroke (Simats et al., 2024).

Epigenetic modifications, the alteration in gene expression via changes of chromatin structure and accessibility, form the foundation of TRIM (Berger et al., 2009). Key histone modifications, including histone 3 lysine 4 mono-methylation (H3K4me1), which is typically enriched at enhancer regions, histone 3 lysine 27 acetylation (H3K27ac), which marks active enhancers and some promoters, trimethylation (H3K4me3), which localizes predominantly at gene promoters near transcription start sites, along with decreased DNA methylation at the promoters of genes associated with immune pathways, act as important epigenetic markers following primary stimulation (Beacon et al., 2021; Das et al., 2019; Verma et al., 2017; Wang and Helin, 2025). These changes facilitate the transformation of pro-inflammatory gene loci from a quiescent state to a more accessible ‘trained’ state (Fanucchi et al., 2021; Ghisletti et al., 2010; Novakovic et al., 2016). Recent studies indicate that BCG-induced trained immunity in bone marrow–derived macrophages (BMDMs) relies predominantly on H3K4me1 at enhancers, rather than H3K4me3 or H3K27ac, since H3K4me1 is maintained long-term after primary stimulation and closely correlates with the sustained transcriptional and functional reprogramming of the cells (Sun et al., 2023). However, since different stimuli can shape the epigenetic landscape in a cell type–specific way, further studies are needed to determine whether particular epigenetic modifications are commonly required for the induction of innate immune memory.

Additionally, alterations in cellular metabolism is another typical feature of TRIM, shown as a shift from low biosynthetic activity and energy requirements primarily supported by oxidative phosphorylation and fatty acid oxidation to the upregulation of both aerobic glycolysis and oxidative phosphorylation (Arts et al., 2016a; Bekkering et al., 2018; Mitroulis et al., 2018). More detailed insights into the epigenetic and metabolic mechanisms behind TRIM in innate immune cell is reviewed by Stephanie Fanucchi’s previous publication (Fanucchi et al., 2021).

## Cellular constituents of TRIM in brain disease

### Microglia

Microglial cells are the resident macrophages of the central nervous system (CNS). Different from the bone marrow-derived myeloid cells, microglial cells are known to be derived from the yolk sac during the embryonic stage and mainly maintain through cell proliferation, with a minimal contribution from the circulatory monocytes (Ginhoux and Garel, 2018; Hoeffel et al., 2015; Kierdorf et al., 2013). Unlike most other hematopoietic lineages, microglial cells are found to be long-lived, on average 4.2 years old, and renew slowly, with a median turnover rate of 28% per year in the human brain (Ajami et al., 2007; Réu et al., 2017). They perform a wide range of critical functions to maintain brain health and homeostasis via constantly monitoring the CNS environment, detecting and clearing pathogens, and regulating the neuronal activity (Béchade et al., 2013). In response to acute stimuli, such as injury or infection, microglia undergo rapid morphological and functional changes, a process termed microglial activation (Woodburn et al., 2021). By contrast, under certain conditions or following prior insults, microglia may enter a state of priming, in which they retain a resting morphology but are reprogrammed to mount exaggerated responses to subsequent challenges (Perry and Holmes, 2014).

Recent research has revealed the capacity of microglia to develop TRIM, which enables microglia to enter a persistent primed state, characterized by heightened responsiveness to subsequent stimuli (Haley et al., 2019; Martins-Ferreira et al., 2021; Neher and Cunningham, 2019). For example, peripheral stimulation with intraperitoneal lipopolysaccharide (LPS) injection in mice has been shown to induce TRIM in microglia, shown by epigenetic marks at active enhancers for inflammation-related pathways, which exacerbates local pathology in the CNS. Mechanistically, TRIM induction in microglia after LPS stimulation requires transforming growth factor-β–activated kinase 1 (Tak1), a regulator of NF-κB, JNK, and ERK1/2 signaling, as well as histone deacetylases 1 and 2 (Hdac1/2), which are key modulators of epigenetic reprogramming (Wendeln et al., 2018). Sterile injuries, such as microinfarcts, can also trigger TRIM in microglia, evidenced by long-lasting H3K4me3 modification on the promoters of TNF-α, IL-6, IL-1β, and IL-10 (Feng et al., 2024). In vitro studies further support this notion that the first priming with a specific concentration range of LPS enables microglia an increased inflammatory response upon the secondary stimulation, seen as increased phagocytic capacity and mRNA level of pro-inflammatory cytokines (Huang et al., 2023b; Lajqi et al., 2019). Epigenetic modification, especially H3K27ac and H3K4me3 modification on the promoters of several proinflammatory cytokines was found to underly the microglial training after LPS (Feng et al., 2024; Huang et al., 2023b).

### Border-associated macrophages

Border-associated macrophages (BAMs) are a distinct subset of brain-resident myeloid cells located at the borders of the CNS, including the choroid plexus, meninges, and perivascular spaces (Sun and Jiang, 2024a). Similar to microglia, BAMs are mostly derived from early erythro-myeloid progenitors in the yolk sac (Mrdjen et al., 2018). While perivascular and subdural macrophages are long-lived and do not rely on replenishment from bone marrow-derived monocytes, choroid plexus BAMs are gradually replaced by bone marrow-derived precursors (Utz et al., 2020; Van Hove et al., 2019). Recent work further shows that dural macrophages include an extrasinusoidal subset that is also replenished by monocytes (Amann et al., 2024). Aside from detecting and clearing hazardous substances through phagocytosis and antigen-presenting function, BAMs play a crucial role in preserving blood-brain barrier (BBB) integrity through interacting with the endothelial barrier (Wen et al., 2024). Following pathological insults, BAMs are increasingly replenished by CCR2^+^ monocytes, which can differentiate into long-lived BAMs (Wang et al., 2024). Although the role of TRIM in BAMs activity and their involvement in brain diseases remains unexplored, the significant contribution of bone marrow-derived precursors to the BAM population suggests that training could occur, influencing their long-term phenotype and function during CNS pathology. Given their critical role in brain homeostasis, further research is essential to elucidate the potential involvement of BAMs in neurological disorders.

### Myelopoiesis and monocytes

Besides brain-resident immune cells, bone marrow-derived leukocytes are another important source of the pro-inflammatory responses in brain disorders. It has been established that TRIM can occur not only in mature myeloid cells within periphery immune compartments, such as brain, referred to as ‘peripheral TRIM’, but also in long-lived HSPCs in the bone marrow, the central immune compartment, known as ‘central TRIM’ (Kaufmann et al., 2018).

Our previous study has shown that during acute ischemic brain injury, hematopoietic progenitors in the bone marrow shift toward myelopoiesis, a process that persists at least several months after the initial insult (Simats et al., 2024). Under normal conditions, monocytes are absent from the brain parenchyma. However, during CNS pathology, peripheral monocytes can be mobilized, breach the BBB, and infiltrate into the brain. In a mouse model of traumatic brain injury (TBI), long-term alterations in the epigenetic landscape of bone marrow HSPCs were observed. Transplantation of bone marrow cells from post-TBI mice into healthy aged recipients led to persistent transcriptional and functional changes in peripheral myeloid cells, indicating central TRIM post-TBI. These bone marrow-derived alterations also drove sustained neuroinflammation and neurological deficits in the chimeric mice (Ritzel et al., 2024), supporting a significant role of central TRIM in CNS pathology.

### Non-immune cells

Besides classical brain immune compartments, various non-immune cells, such as endothelial cells, oligodendrocytes, and astrocytes, have been shown to possess immunological functions. Although the precise molecular mechanisms behind the ‘innate immune’ functions are not fully understood, their ability to express various pattern-recognition receptors (PRRs) like Toll-like receptor (TLRs) and NOD1/2 suggests those cells can respond to both endogenous and exogenous stimuli (Farina et al., 2007; Kapil et al., 2012; Opitz et al., 2009). Upon pathological insult, those non-immune cells could be activated, undergo morphological changes, and produce pro-inflammatory cytokines. Notably, they also exhibit characteristics of TRIM. For instance, treating endothelial cells with oxidized oxLDL enhanced their inflammatory cytokine production upon secondary stimulation, which is also driven by metabolic and epigenetic reprogramming, suggesting that TRIM-like mechanisms extend beyond the classical immune cells (Sohrabi et al., 2020).

Astrocytes are the most abundant glial cells present throughout the brain. They play critical roles in maintaining CNS homeostasis. During the quiescent state, they form the glia limitans around blood vessels, restricting the access of immune cells to the CNS parenchyma. Besides, astrocytes are responsible for the metabolic support of neurons, glycogen storage, export of lactate and uptake of neurotransmitters, including glutamate (Freeman, 2010; Patani et al., 2023). In the healthy adult mouse brain, astrocytes are largely long-lived with minimal turnover in regions, such as the cortex, as shown by a previous study (Tay et al., 2017), whereas limited ongoing astrogenesis occurs in niche-specific sites like the dentate gyrus and hypothalamus through local proliferation (Schneider et al., 2022). Recent research has shown that astrocytes can exhibit TRIM characteristics. In both in vivo and in vitro experiments, astrocytes demonstrated heightened responsiveness to secondary stimulation following dual exposures to IL-1β and TNF. This increased reactivity was linked to p300-driven H3K27ac, regulating pathways related to metabolism, NF-κB signaling, and inflammation (Lee et al., 2024). Given their long lifespan and low turnover rate, astrocytic epigenetic memory may contribute to the progression of chronic neurological disorders.

### TRIM in acute and chronic neurodegeneration

Advances in medical technology and economic growth have contributed to increased life expectancy, which in turn has led to a growing elderly population and a corresponding rise in the prevalence of primary and secondary age-related neurodegenerative diseases (Erkkinen et al., 2018). These disorders encompass a wide range of pathological conditions, characterized primarily by the progressive loss of neuronal structure and function in the brain and peripheral nervous system (Lamptey et al., 2022). Despite significant progress in understanding the pathogenesis of neurodegenerative disorders over recent decades, treatment options remain limited and are largely focused on symptom management and slowing disease progression. A deeper understanding of the mechanisms driving the onset and progression of these disorders is, therefore, essential.

**Figure 1.**
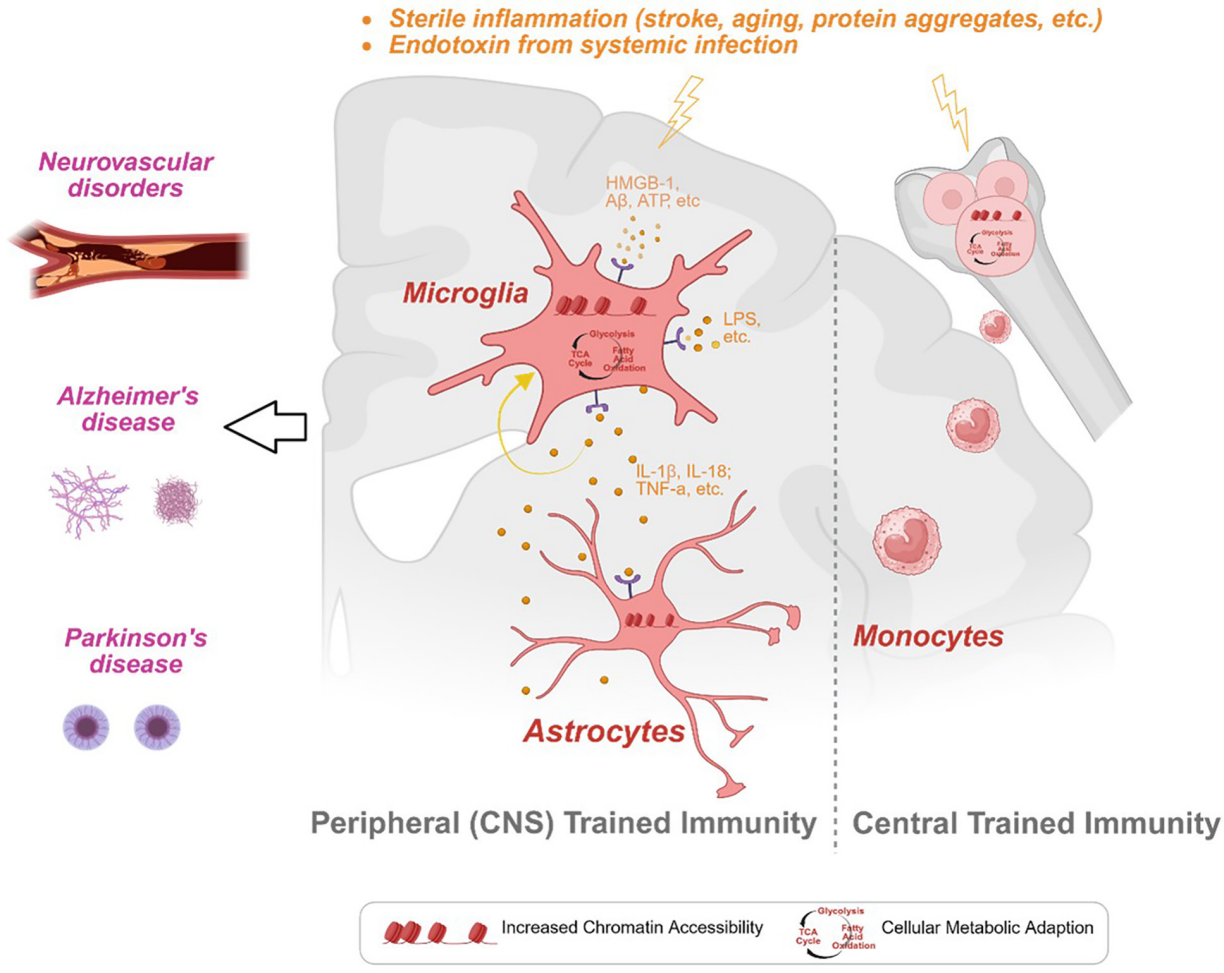
Trained immunity (TRIM) in central nervous system (CNS) innate immune cells and its role in acute and chronic brain disorders. This figure provides an overview of TRIM in CNS innate immune cells, including microglia, astrocytes, and peripheral bone marrow-derived monocytes, and their contributions to neurodegenerative disease progression. The left panel highlights common neurodegenerative conditions, such as Alzheimer’s disease, Parkinson’s disease, cerebral small vessel diseases, and ischemic stroke. The central panel focuses on microglia and astrocytes, key CNS-resident immune cells, showing their responses to training stimuli like high-mobility group box 1 (HMGB1), amyloid-beta (Aβ), lipopolysaccharide (LPS), and proinflammatory cytokines (e.g. IL-1β, IL-18, TNF-α). These exposures lead to epigenetic modifications (e.g. H3K4me3, H3K27ac) and metabolic reprogramming (e.g. enhanced fatty acid oxidation) that reinforce trained phenotypes. The right panel represents monocytes in the periphery, emphasizing their epigenetic changes (e.g. H3K4me3) and increased aerobic respiration. Together, these adaptations contribute to sterile inflammation, which can be triggered by aging, stroke, or systemic infection and perpetuate neurodegeneration by amplifying inflammatory responses in the CNS.

Recent studies have highlighted potential training characteristics in the innate immune cells of the CNS. This review synthesizes current findings on the relationship between TRIM and neurodegenerative disorders, with a particular focus on its potential contributions to the development and progression of primary and secondary neurodegenerative pathologies (Figure 1).

### Ischemic stroke

Ischemic stroke is a significant medical issue worldwide, being the third most frequent cause of disability among survivors and the second leading cause of dementia and death (Katan and Luft, 2018). It occurs when the blood supply to a part of the brain is blocked or severely reduced, leading to irreversible neuronal damage primarily caused by necrotic cell death. Following an ischemic event, microglial cells become activated within minutes. This activation occurs through various pathways, including their interaction with DAMPs, which are passively released from dying neurons and non-neuronal cells in the ischemic brain (Gülke et al., 2018).

Besides neuroinflammation, stroke also leads to systemic inflammatory response (Simats and Liesz, 2022). Post-stroke immune responses follow a multiphasic trajectory: initially, DAMPs entering the circulation trigger acute peripheral immune activation, which is followed by a phase of systemic immune suppression, termed stroke-induced immunosuppression (SIIS), characterized by lymphopenia, reduced NK cell counts in blood and spleen, and a shift from T-helper (Th)1 toward Th2 polarization (Faura et al., 2021). In the innate compartment, SIIS is shown by functional impairments, such as reduced respiratory burst activity in neutrophils and monocytes (Ruhnau et al., 2014).

In the chronic phase, however, stroke has been shown to induce central TRIM through reprogramming HSPCs and bone marrow-derived monocytes, thus leading to chronic heart dysfunction (Simats et al., 2024). After a stroke, circulatory monocytes could infiltrate into the brain lesion through the disrupted BBB (Ley et al., 2007; Mo et al., 2013). In addition, the number of bone marrow-derived monocytes/macrophages in the perilesional area gradually increases after stroke, which lasts at least until 6 m (Garcia-Bonilla et al., 2016; Kato et al., 2021). CCR2^+^ monocytes are known to be involved in the progression of neuropathological processes and cognitive dysfunction after myocardial infarction via CCR2-dependent recruitment to the CNS (Thorp et al., 2024). Thus, though no direct evidence, it’s plausible that the infiltrated monocytes, which are reprogrammed after stroke, might contribute to the various detrimental sequela, such as vascular dementia.

Beyond the training of the BM HSPCs and their potential contribution to periphery sequela, stroke-induced neuroinflammation can persist into the chronic phase. Microglial activation in the peri-infarct region has been observed to last for at least several months post-stroke, shown by characteristic inflammatory transcriptional changes as well as corresponding morphological changes (Sanchez-Bezanilla et al., 2021; Weber et al., 2025). Though a comprehensive analysis of microglial epigenetic changes throughout the different phases of stroke recovery is still missing, one recent study demonstrated that microglia undergo metabolic reprogramming during the chronic phase in a stroke mouse model (Loppi et al., 2023), which indicates key traits of innate immune memory might underly the prolonged and unsolved activation of microglia after ischemic insults.

Additionally, neuroinflammation seems to be spread to the remote area at later timepoints after stroke (Gerhard et al., 2005; Stuckey et al., 2021; Walter et al., 2015). For instance, [(¹¹C)PK11195]-PET imaging revealed secondary microglial activation in the ipsilateral thalamus, which persisted up to seven months after stroke. Histological findings also revealed signs of microglial activation as well as neuron loss from those remote areas (Walberer et al., 2014). Recent studies suggest that inflammation in remote regions plays a central role in post-stroke secondary neurodegeneration (SNDG) — the progressive spread of neurodegenerative processes to distant brain structures. This phenomenon is marked by delayed neuronal loss and reactive astrocytosis in areas, such as the ipsilateral thalamus, substantia nigra, and pyramidal tract following a stroke (Brunelli et al., 2023). Long-lasting neuroinflammation, such as pro-inflammatory cytokine secretion, microglial and astrocyte activation have been detected in secondary damage regions (Block et al., 2005; Kluge et al., 2019; Loos et al., 2003; Schroeter et al., 2006). Detailed studies are needed to map the dynamic epigenetic and metabolic landscape of resident and infiltrated immune cells post-stroke and to understand how these changes correlate with SNDG and other functional outcomes.

### Cerebral small vessel diseases

Cerebral small vessel disease (cSVD) encompasses a wide range of pathological conditions affecting the small arteries, arterioles, venules, and capillaries within the brain. It is a leading contributor to cognitive decline and dementia, characterized by intracranial atherosclerosis with thickening and hardening of intracerebral arterioles (Pinter et al., 2015). It has been well established that inflammation plays a crucial role in the initiation and progression of cSVD (Shoamanesh et al., 2015). Elevated serum levels of inflammatory markers, such as IL-1α and IL-6, have been found to be significantly associated with worse clinical outcomes in cSVD (Staszewski et al., 2019). Recent studies have also highlighted the role of microglial activation in cSVD, particularly in hypertensive arteriopathy, a subtype of cSVD commonly linked to chronic high blood pressure (Low et al., 2020).

Monocytes have also been implicated in the pathophysiology of cSVD. A recent clinical study of patients with mild-to-severe cSVD demonstrated a shift of monocytes toward the CD14^++^ CD16^+^ intermediate subset in individuals with rapidly progressing disease, suggesting reprogramming of myelopoiesis. Moreover, basal and stimulated production of inflammatory cytokines in peripheral blood mononuclear cells (PBMCs) was correlated with cSVD progression, indicating functional reprogramming of circulating monocytes (Noz et al., 2018). Transcriptome analysis further revealed the enrichment of several inflammation-related pathways in monocytes from patients with cSVD progression (Noz et al., 2021). Although comprehensive epigenetic and metabolic profiling of peripheral and bone marrow immune compartments is still lacking, current functional and transcriptional data suggest that ‘trained’ monocytes may play a critical role in contributing to the development and progression of cSVD.

## Alzheimer’s disease

Sporadic Alzheimer’s disease (AD) is the most prevalent form of dementia among the elderly and remains lack of effective treatments, characterized by intracellular neurofibrillary tangles composed of hyperphosphorylated tau protein and extracellular amyloid-beta (Aβ) plaques (Botella Lucena and Heneka, 2024). In recent years, neuroinflammation has emerged as another critical hallmark and driver of AD pathogenesis. Microglial activation, an early event in AD, has been observed to play complex and dynamic roles in the disease progression (Cagnin et al., 2001). On one hand, activated microglia may exhibit protective effects by promoting Aβ clearance (Sun et al., 2024c). On the other hand, they can contribute to neurodegeneration by releasing toxic substances that damage neurons and mediate synapse loss through complement-dependent mechanisms (Hansen et al., 2018; Hong et al., 2016).

Microglial cells in early AD have also been shown to exhibit features of TRIM. Aging, the leading risk factor for AD, is associated with extensive epigenomic remodeling at the level of DNA and histone modifications, which further contributes to disease susceptibility (Benayoun et al., 2015; Li et al., 2023). Additionally, systemic inflammatory responses have been recognized as another potential risk factor for AD. For instance, infection with Porphyromonas gingivalis, a chronic oral pathogen, has been shown to drive elevated Aβ production, tau pathology, and neuronal degeneration (Dominy et al., 2019). Likewise, evidence from both clinical and experimental studies suggests that Helicobacter pylori infection may influence the course of AD (Doulberis et al., 2018). Moreover, epidemiological findings indicate that higher levels of systemic inflammatory markers, such as glycoprotein acetyls (GlycA), are associated with brain atrophy and a faster progression of cognitive decline in late mild cognitive impairment (Liang et al., 2024). Systemic inflammation can imprint epigenetic reprogramming on microglia. This reprogramming leads to a TRIM phenotype, characterized by an amplified release of pro-inflammatory mediators upon following challenge, which can exacerbate AD neuropathology (Wendeln et al., 2018). Aβ itself can act as a DAMP, binding to receptors, such as CD14, CD36, and TLRs, thereby activating the NLRP3 inflammasome (Halle et al., 2008). This activation promotes the release of pro-inflammatory cytokines like IL-1β and IL-18, which are key elements of the innate immune response in AD (Halle et al., 2008; Heneka et al., 2013). Studies in both AD mouse models and human AD patients have revealed epigenetic modifications affecting immune-related genes, supporting the hypothesis that microglia acquire trained immunity–like features in AD (Mastroeni et al., 2015; Phillips et al., 1989).

Recent research has identified Aβ aggregates in the plasma of patients with mild cognitive impairment (MCI), indicating potential activation of systemic immunity at early AD by circulating amyloid (Juul-Madsen et al., 2024). Notably, one recent research reported that bone marrow stem cell transplantation from donors with a pathogenic mutant allele of AD can transfer CNS pathologies to healthy recipients, suggesting the contribution of abnormal HSPCs to AD pathogenesis within the brain (Singh et al., 2024). In addition, wild-type (WT) HSPC transplantation rescued the AD phenotype in 5xFAD mice and that transplantation may prevent microglia activation, with a significant decrease in gene expression related to ‘disease-associated microglia’ in the cortex (Mishra et al., 2023). Similarly, young bone marrow transplantation was capable of reversing the cerebral Aβ plaque burden, neuronal degeneration, neuroinflammation, and behavioral deficits in aged APP/PS1 mice (Sun et al., 2024b), revealing a potential central TRIM feature of the bone marrow in AD and their critical role in the development and progression of pathology.

Moreover, monocyte-derived cells have been found to invade brain parenchyma in human AD hippocampus (Muñoz-Castro et al., 2023). Like microglia, monocytes from MCI and AD patients exhibit a pro-inflammatory phenotype in early AD, shown by increased cytokine production in response to LPS stimulation (Bossù et al., 2008; Guerreiro et al., 2007). Meta-analyses confirm elevated levels of pro-inflammatory cytokines in the blood or CSF from patients with early or mild forms of AD, further implicating the contribution of periphery inflammation to the disease’s progression (Boccon-Gibod, 1988; Popp et al., 2017). However, as advanced disease stages, peripheral monocytes become less responsive to stimulation, indicating the development of immune exhaustion or potential innate immune tolerance following repeated and unresolved inflammatory challenges (Faktor et al., 1992; Motta et al., 2007).

In summary, TRIM in microglia and monocytes appears to significantly impact the development and progression of AD. However, the precise mechanisms, including the dynamic epigenetic and metabolic profiles involved, remain largely unexplored. Future research into these areas is essential to deepen our understanding of the immune triggers in AD and to identify potential therapeutic targets.

### Parkinson’s disease

Parkinson’s disease (PD) is a complex and progressive neurodegenerative disorder that impacts both the CNS and peripheral organs. It is primarily characterized by the degeneration of dopaminergic neurons in the substantia nigra pars compacta, resulting in motor dysfunction. A key pathological hallmark of PD is the accumulation of intracellular aggregates of α-synuclein (α-syn), which form Lewy bodies. These aggregates exhibit prion-like behavior, spreading from cell to cell through release from neurons and uptake by neighboring structures, including axons and dendrites (Dickson, 2018; Ye et al., 2023). The development of PD is influenced by a combination of genetic and environmental factors, with emerging evidence highlighting the critical role of the immune system in both its onset and progression. Microglial activation, observed in the early stages of PD, is implicated in driving disease progression (Garcia et al., 2022; Imamura et al., 2003; Lavisse et al., 2021; Ouchi et al., 2005). Specifically, the activation of the NLRP3 inflammasome in microglia triggers caspase-1 activation and the subsequent release of pro-inflammatory cytokines, such as IL-1β and IL-18, contributing to neuronal damage (Anderson et al., 2023).

In addition to promoting inflammation, microglia play a crucial role in clearing α-syn aggregates through phagocytosis, which is mediated by receptors, such as complement receptor 4 (CR4) and TLR4. These receptors influence the propagation and seeding of α-syn aggregates, underscoring the pivotal role of microglia in early PD-related neurodegeneration (Fellner et al., 2013; Garcia et al., 2022; Juul-Madsen et al., 2020). Although direct evidence for microglial TRIM in PD is currently lacking, several studies suggest the presence of epigenetic modifications that could contribute to disease development. These include altered DNA methylation of pro-inflammatory genes, such as iNOS and TNF-α, as well as aberrant histone methylation patterns (Pieper et al., 2008; Searles Nielsen et al., 2015; Toker et al., 2021). Such modifications point to potential training-like characteristics in microglia, which may influence PD pathogenesis and progression. Further research into the epigenetic regulation of microglia could reveal novel insights into their role in PD.

Systemic immune dysregulation is another prominent feature of PD (Cossu et al., 2023; Huang et al., 2023a). For instance, numerous clinical data have revealed that patients with inflammatory bowel disease (IBD) have a significantly elevated risk of developing PD (Park et al., 2019; Zhu et al., 2022). Peripheral innate immune cells, particularly monocytes, exhibit distinct alterations in PD patients (Tian et al., 2022). These monocytes show diminished response to LPS and fibrillar α-syn stimulation, evidenced by reduced production of pro-inflammatory cytokines (Su et al., 2022). Notably, monocytes from early-stage PD patients have been shown to exhibit altered phagocytic capacity depending on culture conditions, with greater phagocytosis observed under more physiologically relevant conditions, highlighting the importance of context in assessing immune function in PD (Wijeyekoon et al., 2018).

In summary, while direct evidence of epigenetic and metabolic changes is still lacking, the significant changes in both peripheral and local innate immunity suggest they may be shaped by various triggers, highlighting the need for further detailed research.

## Key open questions and research directions

### TRIM in human patients with brain disease

TRIM is a double-edged sword. On the one hand, the Bacillus Calmette–Guérin (BCG) vaccine, originally developed for tuberculosis prevention, is the most extensively studied example of TRIM in clinical practice and has been shown to improve responsiveness to influenza vaccination (Garly et al., 2003; Leentjens et al., 2015). Beyond its protective effect against diverse pathogens, BCG-induced TRIM has been used successfully in cancer treatment, particularly in managing high-grade non-muscle invasive bladder cancer and carcinoma in situ, where it lowers recurrence and disease progression (Gandhi et al., 2013; Miyake et al., 2022). Moreover, recent studies indicate that BCG treatment can modulate microglial activity in the aging brain by inducing innate immune training through epigenetic reprogramming, thereby enhancing myelin debris clearance and promoting remyelination (Tiwari et al., 2024). These outcomes demonstrate the potential of BCG-induced TRIM to treat diverse conditions, suggesting broader applications in medicine.

However, TRIM can also have detrimental consequences. For instance, central TRIM is evident in cSVD patients, where blood innate immune cells from those with significant disease progression show heightened inflammatory cytokine production in response to Pam3CysK4 and transcriptomic upregulation of inflammation-related pathways (Noz et al., 2018; Noz et al., 2021). Similar changes are also found in AD and PD, where patients’ monocytes show a trained pro-inflammatory state at the early stage (Bossù et al., 2008; Guerreiro et al., 2007; Wijeyekoon et al., 2018). Additionally, post-mortem brain studies in AD show epigenetic changes, including a nuclear loss of the histone mark H3K4me3 accompanied by its progressive cytoplasmic accumulation, which occurs prior to detectable tau pathology and may contribute to the persistent inflammation characteristic during the early stages of disease progression (Mastroeni et al., 2015).

As previously discussed, TRIM plays a dual role, providing significant protection in infectious and tumor-related diseases while also contributing to the development and progression of various other conditions. Therefore, further research, particularly in human patients, is essential to better understand how to utilize its potential for disease prevention and treatment, as well as to clarify its role in disease pathology. A critical challenge in translational research lies in the differences between human patients and the experimental animal models typically used in TRIM studies. Unlike the young and naïve mice in these experiments, human patients undergo significant age-related changes and are exposed to various ‘training’ stimuli at different doses. This cumulative exposure may leave a more complex set of epigenetic marks on innate immune cells, raising questions about whether TRIM contributes as significantly to disease pathology in humans as it does in animal models. Additionally, as the disease pathology progresses, the immune state may shift from a trained to a tolerant phenotype, potentially driven by immune exhaustion (Cunha and Perazzio, 2024). Thus, addressing the dynamic adaptations of the immune system in response to sequential stimuli over an extended time frame may help to fill the gap between animal studies and the pathological processes in human patients.

## Diversity and dynamics of TRIM

As discussed before, TRIM exhibits functional diversity. While it enhances host defense against pathogens and tumor cells, it can also exacerbate hyperinflammation under chronic or acute sterile conditions. A critical factor influencing these outcomes may lie in the state of immune cells during the resting phase. For example, BCG-trained monocytes acquire epigenetic marks but do not show transcriptional and functional changes in a resting state (Arts et al., 2018; Moorlag et al., 2024), suggests BCG’s potential safety as a non-specific vaccine against microbial infections (Moorlag et al., 2020b). Our recent findings have identified that stroke-induced TRIM creates a persistent pro-inflammatory state, marked by epigenetic modifications as well as transcriptional changes of the HSPCs (Simats et al., 2024). Similarly, other studies have shown that severe COVID-19 infection and a Western diet can induce TRIM, leading to long-lasting changes in hematopoiesis as well as extensive transcriptomic and epigenetic alterations, even in the absence of additional stimulation (Cheong et al., 2023; Christ et al., 2018). Overall, the outcomes of TRIM are shaped by mechanisms, such as metabolic rewiring and epigenetic modifications, which are influenced by the nature of the initial triggers.

In addition, the same agent can produce opposite outcomes depending on dosage. For example, LPS-induced TRIM in lung tissue provides protection against pneumococci and SARS-CoV-2, while in the heart, LPS-induced tolerance via interferon signaling can mitigate isoproterenol-induced injury (Kang et al., 2024; Lim et al., 2024). The intensity of stimuli is crucial in determining immune cell fate. In vitro studies show that ultra-low doses of LPS promote TRIM, while high doses induce immune tolerance (Ifrim et al., 2014; Lajqi et al., 2019). Additionally, different routes of BCG administration can produce varied immune memory phenotypes in innate cells, likely due to differences in concentration in target organ (Andualem et al., 2023), suggesting dose-dependent pathway activation.

The functional outcomes of trained innate immune cells are significantly influenced by their interactions with non-innate immune cells. For example, previous studies have demonstrated that BCG-trained macrophages can exacerbate dermal fibrosis in an HOCL-induced systemic fibrosis mouse model by activating fibroblasts (Jeljeli et al., 2019). Similarly, our research has shown that trained monocytes can activate fibroblasts in distant organs, contributing to inflammatory cardiac disorders (Simats et al., 2024). Furthermore, recent findings reveal that sepsis-trained lung-resident macrophages can promote T cell residency, leading to suppressed tumor growth (Broquet et al., 2024). These examples highlight how trained immune cells can modulate the function of other cell populations, thereby influencing the progression of various diseases.

In summary, TRIM varies widely based on agonists and receptors, doses, pathways, and cell-cell interactions. Current understanding of how these diverse outcomes is generated remains limited. Elucidating these mechanisms in greater detail is essential for a more comprehensive understanding of disease development and progression, as well as for designing novel immune-based therapies for neurological disorders.

### Potential therapeutic target based on TRIM

TRIM not only provides a mechanistic explanation for pathological disease progression but could also offer novel targets for treating diverse brain diseases. We and others have demonstrated that blocking ligand-receptor interactions during the ‘training’ phase, such as with IL-1β neutralizing antibodies or IL-1β receptor antibodies, can prevent the development of central TRIM upon stimulation (Mitroulis et al., 2018; Simats et al., 2024). Additionally, epigenetic regulators have been shown to influence TRIM by modulating transcriptional activation. For instance, in β-glucan–trained monocytes, metabolic reprogramming drives fumarate accumulation, which inhibits activity of the KDM5 family of histone demethylases and promotes epigenetic remodeling; this effect can be partially reversed by the KDM5 cofactor α-ketoglutarate, thus partially counteract the training effect of fumarate (Arts et al., 2016b). Similarly, β-glucan exposure increases lysine methyltransferase Set7 expression in macrophages, and Set7 inhibition during training attenuates the enhanced cytokine response upon restimulation (Keating et al., 2020). Metabolic modulators, such as rapamycin, torin, AICAR, and 2-deoxyglucose (2-DG), also show the potential to modulate BCG-induced TRIM by inhibition of mTOR and glycolysis, thereby reducing cytokine production upon secondary stimulation (Arts et al., 2016b). Of note, recent research at the single-cell transcriptional level demonstrated significant heterogeneity in TRIM among stimulated human monocytes, as evidenced by substantial expression variability in key inflammatory genes like IL-1β, TNF and CXCL9-11 compared with other genes expressed at a similar level. Notably, IL-1β exhibited the highest level of expression variation (Zhang et al., 2022), underscoring distinct responses to stimulation at the single-cell level. This highlights the importance of monitoring unique ‘trained’ states of individual patients and developing personalized therapeutic protocols.

Altogether, while the concept of TRIM enriches our understanding of brain disorders, much remains to be elucidated to characterize these immune processes across various pathological conditions in detail. Comprehensive research is needed to map the specific epigenetic and metabolic signatures of TRIM in diseases, including stroke, AD, and PD, and other neurodegenerative disorders. This knowledge will enable the development of personalized therapies that target each patient’s unique immune profile and potentially slow the progression of these complex disorders.
